# Disease activity, burden and suffering in patients with ulcerative colitis in the UK cohort recruited into the global ICONIC study

**DOI:** 10.1136/flgastro-2022-102104

**Published:** 2022-06-22

**Authors:** Neeraj Bhala, Ailsa Hart, David Watts, Stephen Lewis, Subrata Ghosh, Chris Hansell, Tariq Ahmad, Stijn Van Haaren, Tom Sensky

**Affiliations:** 1 Institute of Applied Health Research, Queen Elizabeth Hospital Birmingham, Birmingham, UK; 2 IBD Unit, St Mark's Hospital, Harrow, London, UK; 3 Department of Gastroenterology, NHS Forth Valley, Stirling, UK; 4 Department of Gastroenterology, Plymouth Hospitals NHS Trust, Plymouth, UK; 5 College of Medicine and Health, APC Microbiome Ireland, University College Cork, Cork, Ireland; 6 Medical Affairs Department, AbbVie Ltd, Maidenhead, UK; 7 Exeter IBD Group, Royal Devon and Exeter Hospital, Exeter, UK; 8 Exeter IBD Group, University of Exeter, Exeter, UK; 9 Department of Brain Sciences, Faculty of Medicine, Imperial College London, London, UK

**Keywords:** ulcerative colitis

## Abstract

**Objective:**

The Understanding the Impact of Ulcerative Colitis and Its Associated Disease Burden on Patients (ICONIC) was a 2-year, global, prospective, observational study assessing disease burden in adults recently diagnosed (≤36 months) with ulcerative colitis (UC) receiving routine outpatient care, irrespective of disease severity or treatment. A subanalysis was conducted to understand the UK perspective.

**Design/method:**

All eligible consenting patients enrolled in ICONIC from the UK were included in the subanalysis of patient-reported and physician-reported outcomes at baseline and year 2 (Y2).

**Results:**

Sixty-three UK patients were included (mean age 43.4 years, 58.7% female). At baseline and Y2, the mean (±SD) Simple Clinical Colitis Activity Index (SCCAI) scores were 3.6 (±3.3) and 1.5 (±1.5); Patient Modified Simple Clinical Colitis Activity Index (P-SSCAI) were 4.9 (±4.0) and 2.6 (±2.6), respectively. Physician-reported Pictorial Representation of Illness and Self Measure (PRISM) median scores (assessing inverse of suffering) were 3.5 (IQR 2.0–6.8) at baseline and 5.5 (IQR 3.6–6.9) at Y2; patient-reported PRISM scores were 4.7 (IQR 2.6–6.9) and 5.4 (IQR 3.2–8.0), respectively. At baseline, SCCAI and P-SCCAI were strongly correlated (r=0.86, p<0.0001), and patient-reported and physician-reported PRISM scores moderately correlated (r=0.67, p<0.0001). At Y2, moderate correlations were observed (SCCAI vs P-SCCAI: r=0.72, p<0.0001; patient-reported vs physician- reported PRISM: r=0.60, p<0.0001). Rating Form of IBD Patient Concerns scores indicated patients’ greatest concerns were with energy level, having an ostomy bag and effects of medication (baseline scores >3.0).

**Conclusions:**

These findings demonstrated the multifaceted burden of disease in patients recently diagnosed with UC in the UK. Agreement between patients and physicians on disease activity/severity varied according to the instrument used.

**WHAT IS ALREADY KNOWN ON THIS TOPIC**

- Ulcerative colitis (UC) is a chronic, idiopathic, progressive inflammatory bowel disorder that can significantly impact patients’ quality of life.
- The Understanding the Impact of Ulcerative Colitis and Its Associated Disease Burden on Patients (ICONIC) study was a global (33 country), prospective, observational study that described disease burden in patients with recently diagnosed UC.
- Evidence from ICONIC and other studies suggests that physicians and patients may have differing perceptions of disease severity and wider impacts of UC.

**WHAT THIS STUDY ADDS**

- This national subanalysis demonstrated the multifaceted burden of UC in recently diagnosed adults in the UK, with patients reporting impacts across a variety of measures assessing quality of life, illness perceptions, functional status, work productivity, depression and suffering.
- UK patients expressed a variety of disease-related worries and concerns, with the greatest concerns relating to treatment and complications of UC, including energy levels.
- While the degree of agreement between patients and physicians on disease activity/severity varied according to the measure used, the results suggested that UK physicians underestimate some aspects of patient burden.

**HOW THIS STUDY MIGHT AFFECT RESEARCH, PRACTICE AND/OR POLICY**

- A better understanding of the types of burden experienced by UK patients and recognising differences in the appraisal of disease activity/severity between patients and physicians may help improve patient-–physician communication and enable more collaborative discussions about disease symptoms and management.
- Improved awareness of UC-related disease burden experienced by patients from an early stage may help minimise the longer-term negative impacts of UC in areas such as physical and mental health, work productivity and employability, and activity impairment.

## Introduction

Ulcerative colitis (UC) is a chronic, idiopathic, progressive inflammatory bowel disorder characterised by frequent flares followed by periods of remission.^1^ The well-documented burden of UC on patients extends beyond adverse impacts on health-related quality of life (HRQoL), affecting many aspects of daily life including employment opportunities, work productivity and recreational activities.^2–5^ Despite this, physicians often underestimate the burden of UC on patients, and there is limited understanding of patient suffering related to the overall burden of UC.^6^

The Understanding the Impact of Ulcerative Colitis and Its Associated Disease Burden on Patients (ICONIC) study was a 2-year, global, prospective, observational study evaluating the cumulative burden of UC using patient-reported and physician-reported measures of disease activity, HRQoL, work productivity and suffering.^7^ Given the recognised differences in healthcare provision in different countries, a national subgroup analysis has been conducted to provide an understanding of the burden of UC and associated suffering from the UK perspective as part of the larger global study.

## Methods

### Patients

Eligible consenting patients were included in the global ICONIC study if they were aged ≥18 years, diagnosed with UC within the previous 36 months and spoke the language applicable to the study questionnaires, irrespective of treatment or disease activity.^7^ Enrolled patients were evaluated at baseline and every 6 months (±3 months) for 2 years at routine outpatient visits. The present subgroup analysis included all eligible patients enrolled in ICONIC from the UK (n=63 patients from five National Health Service (NHS) hospitals; results from baseline and year 2 (Y2) visits are reported here.

### Data collection and outcomes

Baseline data included demographics, employment status and UC-related disease history (including associated comorbidities and symptoms, associated immune mediated diseases, treatment and hospitalisations). The burden of UC was evaluated at baseline and at each 6-month routine follow-up outpatient visit using a panel of patient-reported and physician-reported validated questionnaires assessing aspects of disease activity, functional status, work productivity, illness perceptions, depression and suffering, as previously described.^7^ Patient-reported outcome measures were the Patient Modified Simple Clinical Colitis Activity Index (P-SCCAI), Short Inflammatory Bowel Disease Questionnaire (SIBDQ), Patient Health Questionnaire (PHQ-9), Rating Form of IBD Patient Concerns (RFIPC), Pictorial Representation of Illness and Self-Measure (PRISM) and Work Productivity and Activity Impairment General Health (WPAI-GH). Physician-reported outcome measures were Simple Clinical Colitis Activity Index (SCCAI) and PRISM. The total score for P-SCCAI was calculated as the sum of the six-item scores (transformed to be equivalent to the SCCAI scoring) with total score ranging between 0 and 19 (higher scores reflect greater symptom severity).^8 9^ The total score for SIBDQ was calculated as the sum of the 10 item scores with the total score ranging between 10 and 70 (higher scores reflect better HRQoL).^10^ The total score for PHQ-9 was calculated as the sum of the nine-item scores with the total score ranging between 0 and 27 (higher scores reflect more severe depressive symptoms).^11^ For the RFIPC, the total score was calculated as the mean of the 25 item scores with the total score ranging between 0 and 10 (higher scores reflect greater worry/concern).^12^ The PRISM is a visual metaphor-based assessment of suffering defined by the Self-Illness Separation score ranging between 0 cm and 9.4 cm (higher scores reflect less suffering).^13^ The WPAI-GH questionnaire measures the effect of general health on four domains (absenteeism, presenteeism and overall work impairment (employed patients only) and total activity impairment), expressed as percentages of impairment (higher scores indicate increased impairment).^14^

### Statistical analyses

Quantitative data (including patient-reported and physician-reported outcome measures) are presented as mean (±SD) or median and IQR (25th percentile–75th percentile) or range. Differences in outcome measures between baseline (visit 1) and Y2 (visit 5) are presented as mean difference (95% CI) (lower bound to upper bound). Categorical data are presented as number (percentage). Spearman’s rank correlation coefficient (r) was used to assess selected correlations between patient-reported PRISM and physician-reported PRISM and SIBDQ, SCCAI, P-SCCAI, RFIPC, PHQ-9 and WPAI-GH, as well as the correlation between patient-reported and physician-reported PRISM and SCCAI scores. Data were analysed using SAS V.9.4.

### Handling of missing data

Percentages were calculated excluding patients with missing values. For SCCAI, P-SCCAI and SIBDQ, if one item was missing, it was substituted with the average score of the non-missing items for calculation of total scores (if >1 item missing, the total scores were not calculated). For PHQ-9 score, if one or two items were missing, they were substituted with the average score of the non-missing items (if >2 items missing, the total scores were not calculated). For RFPIC, missing items were not included in scores and if there were more than six missing items (>25% missing data) the mean total score was not calculated.

## Results

### Baseline demographics, clinical characteristics and disease history

Baseline demographics and disease history for the 63 UK patients included in the present subgroup analysis (mean age 43.4 years, 58.7% female) are presented in table 1.

**Table 1:** Patient demographics and disease characteristics at baseline

Characteristics	All patients (n=63 unless specified)
Female, n (%)	37 (58.7)
Age (years), mean±SD	43.4±15.7
Time since UC diagnosis (days), median (range)*	126 (–7 to 784)
Duration of symptoms prior to UC diagnosis, n (%)	
<1 year	48 (76.2)
1–3 years	8 (12.7)
>3 years	7 (11.1)
Receiving UC treatment at baseline, n (%)	59 (93.7)
Types of UC treatment received at baseline (physician reported), n (%)	(n=64)†
5-aminosalicylic acid or mesalamine	42 (65.6)
Oral systemic steroids	10 (15.6)
Azathioprine or mercaptopurine	4 (6.3)
Aminosalicylates	2 (3.1)
Alternative tumour necrosis factor inhibitors:	
Infusion	1 (1.6)
Biosimilar	1 (1.6)
Sulfasalazine	1 (1.6)
Other (non-listed therapy)	3 (4.7)
Employment status, n (%)	
Employed/self-employed	44 (69.8)
Retired	11 (17.5)
On sick leave	9 (14.3)
Related to UC	6/9 (66.7)
Unrelated to UC	3/9 (33.7)
Unemployed	4 (6.3)
Related to UC	4/4 (100.0)
Unrelated to UC	0 (0.0)
Homemaker	3 (4.8)
Student	1 (1.6)
Duration of sick leave related to UC	(n=6)
<2 months	5 (83.3)
≥2 to ≤4 months	0 (0.0)
>4 months	1 (16.7)
Duration of unemployment related to UC	(n=4)
<6 months	3 (75.0)
≥6 to ≤12 months	1 (25.0)
≥1 UC-related hospital admissions, n (%)‡	13 (20.6)
≥1 UC-related hospital admissions for surgery, n (%)‡	4 (6.3)
≥1 associated comorbid diseases/symptoms§, n (%)	31 (49.2)
≥1 associated immune mediated diseases, n (%)¶	13 (20.6)

*All patients had a confirmed UC diagnosis at baseline—time since UC diagnosis was calculated as the difference between date of UC diagnosis and baseline visit date, and the 15th day was used as the default diagnosis date; therefore, negative values can occur.

†Not mutually exclusive.

‡During the 6 months prior to the baseline visit.

§Relevant comorbid conditions included anxiety/depression, cardiovascular disease, chronic renal deficiency, chronic pulmonary disease, cognitive dysfunction, diabetes mellitus, fatigue, low body weight, polyneuropathy/neuropathy, postural hypertension, skin disease and sleep disorders.

¶Relevant immune-mediated diseases included ankylosing spondylitis, erythema nodosum, hidradenitis suppurativa, primary sclerosing cholangitis, psoriasis, psoriatic arthritis, pyoderma gangrenosum, rheumatoid arthritis and uveitis.

UC, ulcerative colitis.

Of the 63 patients at baseline, 93.7% were receiving UC-related treatment. Nine (14.3%) patients were on sick leave (of these, six (66.7%) attributed sick leave to UC), and four (6.3%) patients were unemployed (all attributed unemployment to UC). In the 6 months before baseline, 20.6% of patients had a UC-related hospitalisation and 6.3% of patients had UC-related surgery.

At baseline, 49.2% of patients had ≥1 associated comorbid diseases and symptoms (table 1). Comorbid diseases and symptoms experienced by ≥30% of all patients were (in order of decreasing frequency) fatigue (34.9%), diabetes mellitus (33.3%), skin disease (33.3%), anxiety or depression (31.7%), cardiac abnormalities/cardiovascular disease (31.7%), chronic renal disease or insufficiency (30.2%), chronic pulmonary disease (30.2%), polyneuropathy/neuropathy (30.2%) and sleep disorders (30.2%, online supplemental table 1). Overall, 20.6% of patients had ≥1 associated immune-mediated disease at baseline.

10.1136/flgastro-2022-102104.supp1 Supplementary data

### Disease activity, disease burden and suffering at baseline and Y2

Physician-reported and patient-reported disease activity at baseline and Y2 are summarised in online supplemental table 2. The degrees of concordance between physician-assessed and self-assessed UC severities for those with both assessments at baseline (n=62) were 53.3% for remission, 66.7% for mild disease, 27.8% for moderate disease and 45.5% for severe disease (online supplemental table 3).

**Figure 1:**
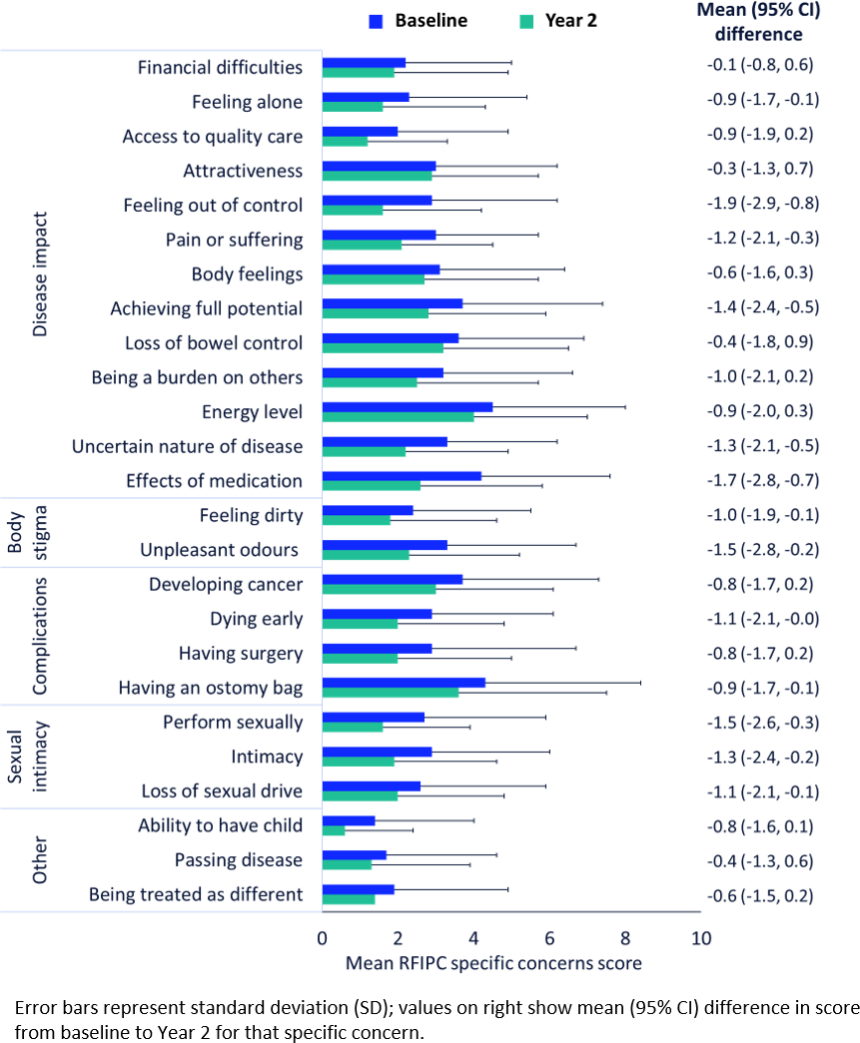
RFIPC scores for specific patient concerns at baseline and Y2, and mean difference in score between baseline and Y2. RFIPC, Rating Form of IBD Patient Concerns; Y2, year 2.

Patient-reported and physician-reported disease activity and suffering at baseline and Y2 and mean differences between baseline and Y2 for the different measures are summarised in table 2. Scores for the specific items of concern in the RFIPC are summarised in figure 1. The highest levels of concern (mean score) at baseline were related to energy level (4.5, SD ±3.5), having an ostomy bag (4.3, SD ±4.1), effects of medication (4.2, SD ±3.4), achieving full potential (3.7, SD ±3.3) and developing cancer (3.7, SD ±3.6). The mean differences in RFIPC scores between baseline and Y2 are summarised in figure 1; general reductions in mean score from baseline were observed at Y2 for all RFIPC items.

**Table 2:** Disease burden and suffering at baseline and Y2

Characteristics*	Baseline	Y2	Mean difference (95% CI)
SCCAI	3.6±3.3† (n=63)	1.5±1.5† (n=37)	−2.2 (−3.1 to –1.3) (n=37)
P-SCCAI	4.9±4.0† (n=62)	2.6±2.6† (n=35)	−2.3 (−3.3 to –1.4) (n=35)
PRISM (physician)	3.5 (2.0–6.8)‡ (n=63)	5.5 (3.6–6.9)‡ (n=34)	1.6 (0.8 to 2.4) (n=34)
PRISM (patient)	4.7 (2.6–6.9)‡ (n=63)	5.4 (3.2–8.0)‡ (n=33)	0.5 (−0.3 to 1.4) (n=33)
SIBDQ	47.0±13.6† (n=63)	56.1±11.1† (n=35)	10.3 (6.4 to 14.1) (n=35)
PHQ-9	7.2±6.6† (n=63)	4.9±5.5† (n=35)	−3.4 (−4.9 to –1.9) (n=35)
RFIPC	2.9±2.3† (n=63)	2.2±2.0† (n=35)	−1.0 (−1.5 to –0.4) (n=35)
WPAI-GH			
Total activity impairment (%)	40.0 (0.0–70.0)‡ (n=60)	5.0 (0.0–30.0)‡ (n=34)	−19.1 (-31.5,–6.7) (n=34)
Total work productivity impairment (%)	30.0 (10.0–60.0)‡ (n=38)	0.0 (0.0–30.0)‡ (n=23)	−25.3 (−43.1 to –7.5) (n=19)
Absenteeism (%)	0.0 (0.0–0.0)‡ (n=38)	0.0 (0.0–0.0)‡ (n=23)	−13.4 (−29.1 to 2.3) (n=19)
Presenteeism (%)	30.0 (10.0–55.0)‡ (n=40)	0.0 (0.0–30.0)‡ (n=23)	−25.2 (−41.6 to –8.9) (n=21)

*Higher scores for SCCAI/P-SSCAI, PHQ-9, RFIPC and WPAI and lower scores for PRISM and SIBDQ indicate greater burden.

†Mean±SD.

‡Median (IQR).

PHQ-9, Patient Health Questionnaire; PRISM, Pictorial Representation of Illness and Self Measure; P-SCCAI, Patient mModified Simple Clinical Colitis Activity Index; RFIPC, Rating Form of IBD Patient Concerns; SCCAI, Simple Clinical Colitis Activity Index; SIBDQ, Short Inflammatory Bowel Disease Questionnaire; WPAI-GH, Work Productivity and Activity Impairment General Health; Y2, year 2.

At baseline, SCCAI and P-SCCAI were strongly correlated (Spearman’s r=0.86, p<0.0001, n=62), and patient-reported and physician-reported PRISM scores were moderately correlated (Spearman’s r=0.67, p<0.0001). There was a moderate negative correlation between SCCAI and physician-reported PRISM score at baseline (Spearman’s r=−0.64, p<0.0001). At Y2, moderate correlations were observed between SCCAI and P-SCCAI scores (r=0.72, p<0.0001, n=34), patient-reported and physician-reported PRISM scores (r=0.60, p<0.0001, n=31), and SCCAI and physician-reported PRISM scores (r=−0.64, p<0.0001, n=34). Moderate correlations were also observed between patient-reported PRISM and other patient-reported outcomes at baseline and Y2 (table 3).

**Table 3:** Correlations between patient-reported PRISM and other patient-reported outcomes at baseline and Y2

Variables	N	Spearman correlation coefficient	P value
Baseline			
PRISM (patient) versus SIBDQ	63	0.71	<0.0001
PRISM (patient) versus PHQ-9	63	−0.56	<0.0001
PRISM (patient) versus P-SCCAI	62	−0.58	<0.0001
PRISM (patient) versus RFIPC	63	−0.71	<0.0001
PRISM (patient) versus WPAI-GH (presenteeism)	40	−0.54	0.0003
PRISM (patient) versus WPAI-GH (absenteeism)	38	−0.38	0.0186
PRISM (patient) versus WPAI-GH (total work productivity)	38	−0.48	0.0022
PRISM (patient) versus WPAI-GH (total activity)	60	−0.65	<0.0001
Y2			
PRISM (patient) versus SIBDQ	33	0.74	<0.0001
PRISM (patient) versus PHQ-9	33	−0.48	0.0044
PRISM (patient) versus P-SCCAI	33	−0.67	<0.0001
PRISM (patient) versus RFIPC	33	−0.82	<0.0001
PRISM (patient) versus WPAI-GH (presenteeism)	22	−0.41	0.0614
PRISM (patient) versus WPAI-GH (absenteeism)	22	–*	–
PRISM (patient) versus WPAI-GH (total work productivity)	22	−0.41	0.0614
PRISM (patient) versus WPAI-GH (total activity)	32	−0.55	0.0012

*Absenteeism was 0% at Y2.

PHQ-9, Patient Health Questionnaire; PRISM, Pictorial Representation of Illness and Self Measure; P-SCCAI, Patient mModified Simple Clinical Colitis Activity Index; RFIPC, Rating Form of IBD Patient Concerns; SIBDQ, Short Inflammatory Bowel Disease Questionnaire; WPAI-GH, Work Productivity and Activity Impairment General Health; Y2, year 2.

## Discussion

This subanalysis of ICONIC provides a current perspective of the diverse burden of UC on various aspects of HRQoL and wider functioning among adults with early disease in the UK.

While there is no gold standard measure used by physicians to assess disease activity in UC, the SCCAI is commonly used as a tool to quantify overall disease activity.^8 9 15^ The mean SCCAI and P-SCCAI scores observed at baseline (both <5) in this UK cohort are consistent with the observation that approximately half of patients were in remission or had mild UC at baseline.^15^ SCCAI scores appeared to improve over time (as might be expected with longer-term management), consistent with the higher proportion of patients in remission or with mild UC at Y2.

While the SCCAI scores were indicative of mild disease, the results also suggest that the burden of UC extends beyond the symptoms and disease activity measures typically routinely used clinically. Consistent with the findings of the ICONIC global study,^7^ the results from patient-reported and physician reported outcomes at baseline suggested that UK patients who had been diagnosed within the last 3 years experienced various UC-related impacts on their HRQoL, mental health and work-related and activity-related productivities. A subset of patients in this UK cohort showed signs of clinically relevant depression at baseline according to the PHQ-9^16^ supporting existing evidence that UC can impact mental health.^3^ The apparent improvement in scores at Y2 (based on mean reduction in scores) may, however, suggest that some of the anxiety and/or depressive symptoms initially experienced by patients are alleviated once they enter the care system.

Descriptive analyses for other questionnaires were also suggestive of general improvements at Y2 for patients newly diagnosed with UC. While the completion rate was lower at this time point, these findings coincide with the wider global ICONIC study^7^ and may reflect effective patient management and/or patient adaptation to the constraints of their chronic condition.

In addition, low absenteeism rates were observed, suggesting that UC was not directly impacting work attendance. Nevertheless, patients appeared to experience relatively high rates of presenteeism and general work and activity impairment at baseline. These results, coupled with the aforementioned depressive symptoms experienced by some patients at baseline, suggest that patients could benefit from additional practical and psychological^17^ support from early in their UC journey in addition to their routine care.

This subanalysis also provided insight into specific worries and concerns experienced by UK patients with UC, a topic that was not fully explored for the full global cohort.^7^ With a maximum possible score of 10, it is somewhat encouraging that the mean total RFIPC score observed was relatively low at baseline and at Y2. However, responses to specific RFIPC items indicated that the domains with the highest levels of concern at baseline (mean scores >3.0) were related to disease impact (energy level, having an ostomy bag, effects of medication and achieving full potential) and complications (developing cancer). The observed concerns about energy levels, coupled with the high prevalence of fatigue reported at baseline, provides further support to the notion that fatigue is an important unmet need for patients with UC.^18^ Consistent with wider findings from the global cohort,^7^ comorbid diseases were also common at baseline, and so wider aspects of the patient’s health may have also influenced their appraisals of disease burden.

Taken together, these results highlight an opportunity for healthcare professionals (HCPs) to engage with patients about their disease symptoms, experiences and worries much earlier in their UC journey. By identifying specific worries and concerns patients may have, it may be possible to alleviate existing fears, as well as to identify particular strategies or techniques that might help patients adapt to their UC and overcome associated anxieties more quickly. This may in turn help limit the wider negative impact (eg, on employment opportunities and social/recreational activities), which may also positively impact depression.

This subanalysis also provides a current perspective on the relationship between patient and physician appraisals of disease activity and suffering in UC in the UK. While responses to the patient and physician version of the SCCAI were highly correlated at both baseline and Y2, the patient-reported mean scores were numerically higher than physician-reported mean scores at both time points. These findings correspond with other research suggesting that while the P-SCCAI can be seen to complement the SCCAI, clinicians may underestimate disease activity and symptom intensity compared with patients.^9^ Conversely, when asked to classify the severity of their disease, 22.7% of the patients classified their disease as being more severe than physicians. These results supplement recent evidence suggesting that physicians place importance on physical symptoms while patients seek support with managing the emotional and mental health impacts of UC. Improved patient education on disease symptoms and open discussion about emotional health may facilitate efficient diagnosis and management.^19^

Consistent with the global ICONIC study,^7^ patient-reported and physician-reported sufferings assessed using PRISM—used for the first time in a UK-based UC population—were moderately correlated with other measures of UC disease activity, depression and worry/concern. PRISM was designed as a proxy for multiple questionnaires and has been used clinically to aid communication between patients and clinicians.^20 21^ Since PRISM can be completed more quickly than other questionnaires, with immediate results, it may be a useful tool for routinely gaining insights into the level of disease suffering experienced by UK patients.

While patient-assessed and physician-assessed PRISM scores were moderately correlated, the physician-assessed scores were suggestive of a general improvement at Y2, whereas patient-assessed PRISM scores were broadly similar at both time points. This finding further implies that patients have different considerations when appraising the burden of their illness compared with physicians.

Overall, these findings suggest there may currently be some disconnect between UK patients’ appraisals of their disease over time and those of the HCPs treating them. In the UK, improving the partnership between physicians and patients is an important priority for the NHS.^22^ A more holistic understanding of the impact of UC and disease burden as reported by patients has the potential to improve patient–physician communication and assessment of the aspects of physical and mental health, work productivity and activity impairment that contribute most towards an individual’s overall well-being.

This subanalysis has some limitations. While the global ICONIC study is one of the largest to assess the burden of UC, the sample size of UK patients was small. Not all patients completed the Y2 assessments, meaning there was insufficient power to enable meaningful statistical comparisons with baseline, and it is unclear if patients who did not complete the questionnaires at Y2 had different experiences. Information on disease extent was limited. A further limitation common to self-reported outcomes, such as questionnaires, is that results are inherently prone to self-presentational and recall biases.

## Conclusions

This subanalysis of the global ICONIC study demonstrates the multifaceted burden of disease in patients recently diagnosed with UC in the UK beyond typical clinical symptoms and disease activity. While in general patients had relatively mild disease, disease burden was reported across various domains including functional status, work productivity, illness appraisals, depression and suffering, suggesting patients experience an array of worries and concerns during the early years following diagnosis. Furthermore, there appears to be some disconnect between patients’ and physicians’ appraisals of disease burden. An improved awareness of the types of burden experienced by UK patients and recognition of differences in the perception of disease activity/severity between patients and physicians may help enable more meaningful discussions about disease symptoms and management from an earlier stage. In turn, this may help to minimise the longer-term negative impacts of UC in areas such as physical and mental health, work productivity and employability, and activity impairment.

## Data Availability

Data are available upon reasonable request. AbbVie is committed to responsible data sharing regarding the clinical trials we sponsor. This includes access to anonymised, individual and trial-level data (analysis datasets), as well as other information (eg, protocols and clinical study reports), as long as the trials are not part of an ongoing or planned regulatory submission. This includes requests for clinical trial data for unlicensed products and indications. These clinical trial data can be requested by any qualified researchers who engage in rigorous, independent scientific research, and will be provided following review and approval of a research proposal and statistical analysis plan and execution of a data sharing agreement. Data requests can be submitted at any time and the data will be accessible for 12 months, with possible extensions considered. For more information on the process, or to submit a request, visit the following link: https://www.abbvie.com/our-science/clinical-trials/clinical-trials-data-and-information-sharing/data-and-information-sharing-with-qualified-researchers.html.
