# Supplementary material for: Disease activity, burden and suffering in patients with ulcerative colitis in the UK cohort recruited into the global ICONIC study
Source: Frontline Gastroenterol. 2022 Jun 22;14(1):25–31. doi: 10.1136/flgastro-2022-102104 (PMC9763636; doi:10.1136/flgastro-2022-102104)
Supplement: Supplementary data [file flgastro-2022-102104supp001.pdf]

Online Supplementary Material

Supplementary table 1. Selected comorbidities at baseline

**Comorbidities at baseline, n (%)**	**All patients** [n=63]
Fatigue	22 (34.9)
Diabetes mellitus	21 (33.3)
Skin disease	21 (33.3)
Anxiety/depression	20 (31.7)
Cardiac abnormalities/cardiovascular disease	20 (31.7)
Chronic renal disease or insufficiency	19 (30.2)
Chronic pulmonary disease	19 (30.2)
Polyneuropathy/neuropathy	19 (30.2)
Sleep disorders	19 (30.2)
Any malignancies	18 (28.6)
Cognitive dysfunction	18 (28.6)
Low body weight (BMI <20 kg/m^2)	18 (28.6)
Postural hypotension	18 (28.6)

BMI, body mass index.

Supplementary table 2. Disease activity at baseline and Year 2

**Characteristics, n (%)**	**Baseline** (n=63)	**Year 2** (n=37)
Physician assessment of UC severity, n (%)		
In remission	16 (25.4)	18 (48.6)
Mild	18 (28.6)	14 (37.8)
Moderate	18 (28.6)	5 (13.5)
Severe	11 (17.5)	0 (0.0)
Missing	0	26
Patient assessment of UC severity, n (%)		
In remission	12 (19.4)	12 (32.4)
Mild	26 (41.9)	13 (35.1)
Moderate	14 (22.6)	11 (29.7)
Severe	10 (16.1)	1 (2.7)
Missing	1	26

Supplementary table 3. Relationship between physician-assessed and self-assessed disease severity at baseline and Year 2

		Physician-assessed disease severity				
		Remission n (%)	Mild n (%)	Moderate n (%)	Severe n (%)	Total n (%)
**Baseline**						
**Self-assessed disease severity**	Remission	8 (53.3)	2 (11.1)	2 (11.1)	0	12 (100)
	Mild	6 (40.0)	12 (66.7)	7 (38.9)	1 (9.1)	26 (100)
	Moderate	1 (6.7)	3 (16.7)	5 (27.8)	5 (45.5)	14 (100)
	Severe	0	1 (5.6)	4 (22.2)	5 (45.5)	10 (100)
	Missing	1	0	0	0	1
	Total	15 (100)	18 (100)	18 (100)	11 (100)	
**Year 2**						
**Self-assessed disease severity**	Remission	4 (50.0)	3 (23.1)	5 (45.5)	0	12 (100)
	Mild	2 (25.0)	5 (38.5)	3 (27.3)	3 (60.0)	13 (100)
	Moderate	2 (25.0)	5 (38.5)	3 (27.3)	1 (20.0)	11 (100)
	Severe	0	0	0	1 (20.0)	1 (100)
	Missing	8	5	7	6	26
	Total	8 (100)	13 (100)	11 (100)	5 (100)	

Shaded cells indicate concordant physician and patient classification.
